# Impact of Dietary Lipid to Carbohydrate Ratio on Elemental Stoichiometric Relationships in Growth Phenotypes of *Ruditapes Decussatus*

**DOI:** 10.1155/anu/9924742

**Published:** 2025-05-16

**Authors:** Kristina Arranz, Iñaki Urrutxurtu, Enrique Navarro

**Affiliations:** Department of GAFFA, Faculty of Science and Technology, University of the Basque Country (UPV/EHU), Aptdo 644, Bilbao 48080, Spain

**Keywords:** growth phenotypes, lipid/carbohydrate proportion, *Ruditapes decussatus*, stoichiometric balances

## Abstract

Understanding the mechanisms of nutrient regulation in bivalves is crucial for optimizing their growth under varying dietary conditions. In the present work, juveniles of the carpet shell clam (*Ruditapes decussatus*) from the same cohort were size-segregated to obtain fast and slow growing phenotypes. These clams were then conditioned to diets presenting a range of lipid/carbohydrate proportions but similar carbon:nitrogen (C:N) ratios. Subsequently, experiments were conducted to determine elemental (C and N) balances in order to achieve the following aims: (a) To identify strategies of homeostatic nutrient regulation in relation to either endogenous (growth phenotype) or dietary factors and (b) to quantify the extent to which stoichiometric adjustments (at both pre- and postabsorptive levels) are accomplished throughout the successive components of elemental balances. The elemental balances of both C and N exhibited higher values under the lipid-rich diets, indicating the presence of nutritional limitations in juvenile clams fed on low lipid/carbohydrate proportion, resulting from a greater digestive imbalance of lipids in diets of low digestibility coupled to limited dietary lipid income. These nutritional limitations were more effectively managed by the fast-growing phenotype, pointing to the importance of enhanced energetic status in sustaining homeostatic nutrient regulation. The stoichiometric coupling between consumed diets and the biosynthetic requirements of growing tissues relied on postabsorptive rather than preabsorptive mechanisms, although notable discrepancies in this regard were observed between conditioning diets.

## 1. Introduction

Bivalve growth and production, and their dependence on variable diet composition, have been the subject of considerable attention for decades [1]. Growth predictions based on retained energy (the SFG approach) have been further refined by the addition of nutrient balance analyses [2–6]. This refinement contributes to a more comprehensive understanding of the dynamics of the physiological processes underlying growth performance. According to the concepts of biological stoichiometry [7], the maintenance of nutrient homeostasis in growing animal tissues is achieved through the concourse of several physiologically based mechanisms, including (1) preingestive selection, whereby, animals preferentially select foods that best meet their needs; (2) adjustments in assimilation patterns, which involve the selective up- or downregulation of specific elements; and (3) metabolism, which allows the elimination of a nutrient when it is in excess. Overall, these mechanisms can compensate for imbalances between biosynthetic requirements and dietary availability of nutrients. The stoichiometric adjustments achieved with diets of variable composition may result in different growth constraints that are difficult to identify from energy flow measurements alone. For example, elemental-based net budgets of fast- and slow-growing juveniles of *Ruditapes philippinarum* were previously analyzed [8] to assess responses to isocaloric monoalgal diets that differed in carbon:nitrogen (C:N) ratio, with the protein:energy ratio representing the differential feature of diet composition. In that work, stoichiometric regulatory processes were found to rely on both preferential N absorption and stoichiometric N release, resulting in a substantial decoupling between energy and net elemental uptake, highlights the importance of nutrient flux computation in understanding these processes.

Besides the C:N ratio, another topic of ongoing interest in aquaculture with regard to food composition is the optimal proportion of lipids to carbohydrates (L:CH ratio) in the diets of bivalves, related to the feasibility of supplementation of microalgal food with low-cost foodstuffs rich in carbohydrates such as wheat or corn flours or commercial yeasts [9, 10, 11, 12]. The majority of papers addressing the impact of diets are developed on the basis of growth experiences that extend for approximately 30–45 days [10, 11, 13–15]. A number of studies have demonstrated the impact of various dietary supplements, including cornstarch [16, 11, 13], cornmeal [11], and wheatgerm [10, 14, 16], on the growth and biochemical composition of tissues in the grooved carpet shell (GCS) clam, *Ruditapes decussatus*. Specifically, substitutions up to 50% of the phytoplankton dose have been shown to yield a growth rate that is analogous to that observed with the control (100% phytoplankton) diet. Similarly, partial substitutions (up to 50%) of *Chaetoceros muelleri* (L:CH ratio = 1.44) by microcapsules (L:CH ratio = 0.21) in the diet of clams *Sinonovacula constricta* and *R. philippinarum* [15] yielded no discernible differences in growth performance or biochemical composition of clams tissues. However, total substitutions resulted in approximately 30% decline in growth rate likely due to the strong reduction observed in the filtration rates of microcapsules compared to those of microalgae. Zhu et al. [17] employed microcapsules with a variable L:CH ratio to further substantiate the overall beneficial impact of increasing the proportion of lipids in the diet on the growth performance of *S. constricta*, with optimal values found at 1:3 for the L:CH ratio.

In addition to the relevance of food supply and its quality, another crucial factor for bivalve hatcheries is the influence of endogenous factors, possibly related to the genetic constitution of individuals (growth phenotypes). This has been demonstrated to exert a substantial influence on the growth rate of bivalves by modulating physiological parameters, particularly those involved in energy acquisition [18–25]. These differences in growth rate within the same cohort have been shown to be sustained for periods of at least 4 months [24, 26]. Consequently, the analysis of interactive effects between these factors and dietary conditions is of the utmost interest, particularly in clams. Previous studies performed by our laboratory have revealed that physiological responses to changes in both the quantity and biochemical composition of the food may be modulated differently in different growth phenotypes [18, 19, 26]. In view of the aforementioned considerations, the investigation of the potential interactions that may emerge from the dietary influences and the interindividual variations in growth could optimize production practices through the selection of lines. Consequently, the present experiments encompassed interindividual comparisons between fast and slow-growing lines, obtained from the same age cohort (for details, see [18]).

In this line, growth rates and related energy balances have been recorded in the GCS clam fed mixtures of microalgae and yeasts of different proportions to achieve a range (0.6–2.2) in the ratios of L:CH in the diet, while C:N ratios were kept constant [18]. Despite the fact that previous studies have demonstrated the feasibility of replacing up to 80% of the diet with yeast for growing *R. decussatus* specimens [27], the results of this study revealed a clear negative effect of increasing the proportion of yeast in the mixture. This negative effect does not appear to be attributable to a dietary lipid limitation alone since the lowest L:CH ratio tested (L:CH ratio = 0.6; corresponding to the 80% substitution of phytoplankton with yeast) was still above the optimal ratio reported in other clam species [15, 17]. In *R. philippinarum*, the reduced feeding rates recorded with the microencapsulated food appear to account for the corresponding low growth performance observed with this diet [15]. In contrast with these findings, the detrimental effects on growth rates of *R. decussatus* were rather based on a strong reduction in the absorption efficiency (AE) of the clams fed the low-lipid diets [18]. This observation points to the importance of a detailed consideration of the digestive balances involved in the differential absorption of dietary components, thereby, substantiating the present approach, in which elemental analysis was incorporated into the characterization of the physiological components of the energy budget. Consequently, C and N balances were determined together with the calculation of C:N ratios and the ratios of both elements to energy in the experimental setup utilized to determine energy balances [18]. These experiments were designed to allow the analysis of both chronic and acute responses to diets differing in composition by a factor of 3.5 in terms of L:CH ratio and were performed independently in fast- and slow-growing phenotypes, thus, extending the range of physiological responses to variable nutritional conditions. In light of the above, the main objectives of this study were: (a) to identify the strategies of homeostatic nutrient regulation in the form of differential processing of elemental components in relation to both nutritional and endogenous factors and (b) to quantify the degree to which stoichiometric adjustments are accomplished throughout the successive components of elemental balances, particularly with regard to their distribution between pre- and postabsorptive processes.

## 2. Material and Methods

Approximately 2600 spat (with an average length ranging from 8 to 16 mm) of the GCS clam, *Ruditapes decussatus*, were procured from the Marine Research Centre (CIMA, Ribadeo, Spain) and transported to our research facilities at an approximate age of 2 months. Two distinct groups of clams were established by segregating the larger individuals (85^th^ percentile) from the smaller ones (30^th^ percentile), which were subsequently classified as fast (F) and slow (S)-growing cohorts, respectively. The initial mean (SD) live weight of these clam groups was determined to be 376.0 mg (71.6) for the fast-growing group and 179.1 mg (54.2) for the slow-growing group, respectively. Prior to conducting the experiments, individuals were confirmed to maintain their F or S status under our uniform laboratory conditions through a pilot growth experiment for 51 days.

### 2.1. Animal Maintenance, Diet Characteristics, and Experimental Design

A detailed account of the dietary composition, animal maintenance, and experimental design can be found in Arranz et al. [18], Materials and Methods, Sections 2.1–2.2). For the purposes of this section, a concise overview will be provided.

The clams from each F and S groups were randomly distributed into two separate feeding tanks and maintained in the same conditions of salinity (34‰) and temperature (17°C). Each tank received a different diet composition (Table 1) at a concentration of 2 mm^3^ L^−1^ (~20,000 cells mL^−1^). The tanks were cleaned of biodeposits and water was changed daily. Diet compositions consisted of mixtures of the microalgae species *Rhodomonas lens* (freshly collected from laboratory cultures) and the baker's yeast (commercial brand Royal from Mondelez International) *Saccharomyces cerevisiae* (dried pellets) in the proportions as indicated in Table 1. Prior to the start of the experiments, diets were characterized in order to determine the proximate biochemical composition as follows: samples of the diet stocks (>1 L) were centrifuged at 4°C and 3700–3800 rpm for 15 min, and the pellets were freeze-dried. Triplicate samples were then used for the independent extraction and quantification of carbohydrates, proteins, and total lipids, employing colorimetric methods (for detailed procedures, see [18]). Both diets exhibited comparable protein content, with the primary distinction in biochemical composition stemming from the markedly higher L:CH ratio observed in the microalgae-rich diet (D1) relative to the yeast-rich diet (D3). This resulted in a 30% increase in energy content per unit of organic weight in D1 compared to D3.

Diets were characterized frequently during the conditioning period and acute exposure experiments, by filtering a known amount of water onto preweighed glass fiber filters (GF/C). The particle concentration and organic content of the suspended food were determined based on gravimetric measurements. Afterwards, the filters were rinsed with ammonium formate (0.9% w/v) to remove any residual salts. The filters were then dried for 24–48 h at 100°C to determine the dry weight and subsequently burned for 6 h at 450°C to determine the ash weight. The total particulate matter (TPM, mg L^−1^) and particulate inorganic matter (PIM, mg L^−1^) were determined through the dry and ash weight, respectively, while the particulate organic matter (POM, mg L^−1^) was obtained as the difference between TPM and PIM. As for elemental analysis (CHN), GF/C filters were rinsed with 50 mL of filtered seawater and immediately frozen at −20°C, lyophilized, and maintained at −20°C until being analyzed. The analyses were conducted at the SGIker facilities (UPV/EHU) by means of a Euro EA Elemental Analyzer (CHNS) from Euro Vector, with acetanilide serving as the standard. Subsets of samples were burned for 6 h at 450°C and subsequently measured in the elemental analyzer to correct for the inorganic C and N fractions. The gravimetric data concerning the concentration of suspended particles (TPM and POM, both in mg L^−1^) and elemental composition (C, H, and N) of D1 and D3 diets are presented in Arranz et al. [18]. The data presented in the aforementioned study revealed no differences in TPM or POM were reported between the acclimation diets. The calculated element ratios (Table 1) were identical for the C:N and slightly different for the C:H ratio, which was approximately 10% higher in D1.

Clams were maintained on the above diets for 30 days to acclimate to feeding conditions before conducting the experiments. These consisted of physiological determinations required in both energy and elemental balances performed on the groups of clams acclimated to D1 and D3. Each acclimation group was measured while feeding the D1 and D3 diets dosed at two different rations: high ration (H: [particles] = 2 mm^3^ L^−1^), which is coincident with the acclimation ration, and low ration (L: [particles] = 1 mm^3^ L^−1^). According to this experimental design, groups of clams were arranged as illustrated in Table 2.

### 2.2. Determinations of the Elemental Balance

The physiological measurements necessary to compute the elemental balances have been previously reported [18]. In brief, five replicates were used per experimental group to determine the clearance rate (CR), AE, ammonia excretion rate (VNH_4_-N), and oxygen consumption rate (VO_2_). Prior to the computation of elemental balances, physiological rates were standardized to a common dry weight of tissue (29.14 mg soft tissue dry weight). The means (SD) of these standard values are presented in Table 2.

In the present work, the values of the physiological parameters presented in Table 2 have been combined with the elemental analysis of the diets and feces to compute the nitrogen and carbon balances as follows:

#### 2.2.1. Ingestion Rates (IRs)

The particulate organic N and C (PON and POC, respectively) were calculated as the product between POM and the proportion of organic N and C present in the diet as follows:

(1) $$\begin{array}{c} \text{PON }\left({\text{mgL}}^{-1}\right)=\text{POM}\times \frac{N\%}{100}. \end{array}$$

(2) $$\begin{array}{c} \text{POC}\left({\text{mgL}}^{-1}\right)=\text{POM}\times \frac{C\%}{100}. \end{array}$$

The IRs of N and C (IR_N_ and IR_C_ (µg h^−1^)) were then calculated by multiplying the CR by the POM of each element:

(3) $$\begin{array}{c} {\text{IR}}_{\text{N}}=\text{PON}\times \text{CR}. \end{array}$$

(4) $$\begin{array}{c} {\text{IR}}_{\text{C}}=\text{POC}\times \text{CR}. \end{array}$$

#### 2.2.2. Absorption Rates (ARs) and Absorption Efficiencies

The ARs of each element (AR_N_ and AR_C_; µgh^−1^) were calculated as the difference between the corresponding ingestion (IR_N_ and IR_C_; µgh^−1^) and egestion rates (ER_N_ and ER_C_; µgh^−1^). These latter were calculated as the product of the organic IR (OIR), the AE, and the proportion of organic N and C in the feces:

(5) $$\begin{array}{c} {\text{ER}}_{\text{N}}=\text{OIR}\times \left(1-\text{AE}\right)\times \frac{\text{N}{\text{\%}}_{\left(\text{feces}\right)}}{100}. \end{array}$$

(6) $$\begin{array}{c} {\text{ER}}_{\text{C}}=\text{OIR}\times \left(1-\text{AE}\right)\times \frac{\text{C}{\text{\%}}_{\left(\text{feces}\right)}}{100}. \end{array}$$

The AE (AE_N_ and AE_C_, in decimal units) was then estimated as the quotient between the AR and IR of each element:

(7) $$\begin{array}{c} {\text{AE}}_{\text{N}}=\frac{{\text{AR}}_{\text{N}}}{{\text{IR}}_{\text{N}}}. \end{array}$$

(8) $$\begin{array}{c} {\text{AE}}_{\text{C}}=\frac{{\text{AR}}_{\text{C}}}{{\text{IR}}_{\text{C}}}. \end{array}$$

#### 2.2.3. Carbon and Nitrogen Loss

Carbon loss due to respiration (*R*_C_; µg C h^−1^) was estimated from the rates of oxygen consumption (mg O_2_ h^−1^), assuming a respiratory quotient (RQ) of 0.9. Respiration rates were obtained with the aid of oximeters through the monitoring of the decline in oxygen concentration in sealed chambers (volume) filled with filtered seawater (0.2 µm millipore membranes). The nitrogen loss due to excretion (E_N_; µg N h^−1^) was assessed through the determination of ammonia production along a 3-h period in flasks filled with 80 mL of filtered seawater (0.2 µm millipore membranes), using the phenol–hypochlorite method [28]. It was assumed that all of the N losses were due to excretion. In both determinations, chambers without animals were employed as controls and were processed in the same way as the rest of the chambers.

#### 2.2.4. Elemental Balances of Carbon and Nitrogen

Elemental balances of carbon (SFG_C_; µg C h^−1^) and nitrogen (SFG_N_; µg N h^−1^) were calculated as the difference between the amount of C or N absorbed and excreted:

(9) $$\begin{array}{c} {\text{SFG}}_{\text{C}}={\text{AR}}_{\text{C}}-R_{\text{C}}. \end{array}$$

(10) $$\begin{array}{c} {\text{SFG}}_{\text{N}}={\text{AR}}_{\text{N}}-{\text{E}}_{\text{N}}. \end{array}$$

### 2.3. Data Analysis

Analyses were performed after testing the data for normality (Shapiro–Wilk) and homoscedasticity (Levene). The statistical significance of differences between treatments (i.e., diet composition and ration or growth category) with respect to elemental balance parameters, was tested through a two or three-way ANOVA, or nonparametric ANOVA on ranks, depending on the specific case being analyzed. Linear regression analysis was used to estimate the relationships between two or more quantitative variables. All of the statistical analysis as well as the elaboration of graphical material was performed by means of the R software, version 4.2.0 [29]. A *p*-value of less than 0.05 was considered to be statistically significant.

## 3. Results

Components of elemental C and N balances were calculated using the physiological rates and efficiencies reported in Table 2, along with Formulas (1)–(10), for the different experimental groups of F and S clams acclimated to diets of different composition and then fed two different composition diets dosed at high and low ration. A summary of the averaged effects of each of the aforementioned variables (based on means of pooled values) on those components is provided in Table 3.

As illustrated in the data presented in this table, the feeding rates (represented by ingestion) of clams acclimated to D1 exhibited an increase relative to those acclimated to D3. However, this led to a reduction in AE, resulting in only minor differences in ARs between both conditioning groups. Similarly, minor effects were observed in the balances (SFG), as the components of energy expenditure were barely affected by diet conditioning.

Regardless of the maintenance diet, exposure of clams to D3 induced a strong positive feeding response leading to an approximately 50% increment in IRs, compared to clams fed D1. However, benefits of this response were fully canceled by the reduced AE experienced by clams when fed on the D3 relative to D1 composition, which resulted in the same AR values for both exposure diets. Similarly, SFG_C_ were consistent across both diets; however, high rates of N excretion with D1 resulted in a reduction of SFG_N_ values.

As for the effects of ration, the decrease in food concentration produced consistent reductions (~50%) in all components of C and N balances and consequent SFGs, while no effects of ration were observed on the averaged values of AE.

Compared to the S group, the F group demonstrated an approximately 50% increase in feeding rates, although this increment declined to approximately 35% for AR and SFG due to slightly higher AEs recorded in slow growers.

The above summary description provides only a limited account of the complexity of effects emerging from the combination of up to four factors affecting the physiological behavior, particularly with regard to the interaction between acute (exposure) and chronic (conditioning) effects of changes in diet composition. To address this complexity, both types of effects were independently approached by means of a series of multiple-factor ANOVA. This tested the statistical significance of (a) the effects of the conditioning diet and growth category (two factors) on the parameters recorded in clams fed the D1 and D3 diets dosed at the food ration (H) used in conditioning (Table 4) and (b) the combined effects of the exposure diet, its composition, and the ration, along with the growth category (three factors), on the parameters recorded in the clam groups that had been acclimated to diets D1 and D3 (Tables 5 and 6). The parameters examined can be classified into two categories: The first category of parameters comprises rates and efficiencies involved in the elemental balances of both elements (C and N). The second category comprises C : N ratios computed for these rates and efficiencies.

### 3.1. Chronic Conditioning Effects

**Figure 1:**
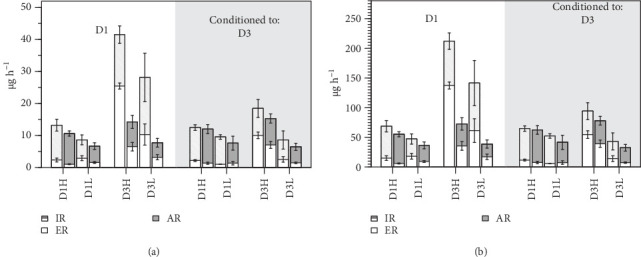
Ingestion (IR), egestion (ER), and absorption rate (AR) mean values for N (a) and C (b) of F (light bars) and S (dark bars) clams subject to all nutritional conditions.

The mean values of the parameters for the complete set of experimental groups are presented in Table 4, along with the results of two-factor ANOVAs that were conducted to compare these means as a simultaneous function of the conditioning diet and the growth group. No significant effects associated with the composition of the conditioning diet were found for any of the components of elemental balances in experiments where clams were fed the D1. However, C:N ratios of feces significantly decreased in the D3 conditioning group, which also entailed significant differences in the C:N ratios for both the rates and efficiencies of absorption. Conversely, significant differences were found between conditioning groups for the ingestion and ERs of clams fed the D3 diet. However, no substantial alterations were evident in the C:N ratios of any of the elemental balance parameters under this exposure diet (Table 4 and Figure 1).

The F clams exhibited higher ingestion, egestion, and ARs of both N and C compared to the S clams, although these differences attained high significance only when fed the D3 diet (Figure 1 and Table 4). The absorption efficiencies of both elements, instead, were significantly higher in the S than in the F clams, irrespective of the exposure diets. Chronic effects of conditioning to either the D1 or D3 diets were neatly different between growth groups, although the affected parameters differed between clams feeding on D1 and D3. The greatest differences were recorded in the exposure to D3, where all acquisition parameters experienced an approximately twofold increase in F clams conditioned to D1 relative to D3, while no such effect was evident in S clams. This complex response is explained by significant effects of both growth group (G) and the interaction with conditioning diet (D^*⁣*^*∗*^^G) for most parameters of the elemental balances and resulting SFG values. The main F vs. S differences during exposure to D1 were documented in the C:N ratios for egestion and absorption, as well as AEs, where significant effects of growth group (G) were recorded. However, the direction of these F vs. S differences shifted between D1 and D3 conditioning groups, as indicated by significant interactions D^*⁣*^*∗*^^G (Table 4).

As a general observation, the C:N ratios decreased sequentially along the successive balance components, from average values of 5.15 for the ingested ration to 4.9 for the absorbed ration and to 4.25 for the final balance or SFG, due to the increase in the C losses relative to N losses both in the feces, and to a greater extent, in metabolic products (the average C:N ratio for metabolic processes was 26.5). Both of these stoichiometric adjustments occurred for each experimental group, although the magnitude of the latter differed between treatments according to differences in metabolic C:N values. This resulted in a greater ratio in clams fed D3 compared to D1 (31.59 vs. 21.55) or conditioned to D1 relative to D3 (31.31 vs. 21.83), while remained similar for the two growth groups (26.47 and 27.29 in F and S clams, respectively).

### 3.2. Acute Effects Induced by Changes in Diet Composition and Ration

Short-term effects of changes in the quality (composition (C)) and quantity (ration (R)) of exposure diets on the components of the elemental balances were analyzed in F and S individuals. To facilitate interpretation of these effects, statistical testing relied on three-factor ANOVAs applied separately to each conditioning group (D1 and D3; Tables 5 and 6). Both the composition and concentration of the diets fed in the exposure experiments exerted significant effects on most acquisition parameters (i.e., ingestion, egestion and ARs and absorption efficiencies of C and N), either considered as isolated factors or through their interaction (C^*⁣*^*∗*^^R). Moreover, these characteristics hold for both groups of conditioning D1 (Table 5) and D3 (Table 6). In general, the rates of nutrient acquisition (both C and N) increased with increasing food concentration (H vs. L rations) and were higher when feeding the diet D3 compared to D1, although this later effect of food composition was much stronger in clams acclimated to D1 compared to D3 (Table 3).

**Figure 2:**
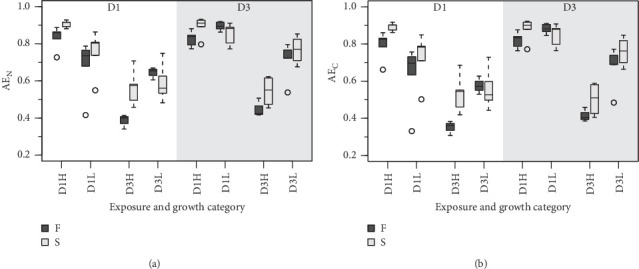
Absorption efficiencies for N (a) and C (b) of F (dark boxes) and S (light boxes) clams subject to all nutritional conditions.

The absorption efficiencies of both N and C (Figure 2) were mainly affected by the composition of the exposure diet. Clams fed the D3 exhibited, on average, an approximately 40% reduction in AEs relative to those fed D1. As previously indicated (Table 3), this tends to offset the effects of overfeeding achieved with D3, resulting in AR differences that are only marginally significant or not significant (Tables 5 and 6). On the other hand, the behavior of AE in response to a change in food concentration (ration) was strongly dependent on diet composition, as evidenced by the high significance of the corresponding interaction term (C^*⁣*^*∗*^^R; Tables 5 and 6). For instance, the effects of increasing the D1 ration on AE were either positive or neutral, while they were clearly negative in the case of D3 (Figure 2), likely due to the greater increases in IRs achieved with this diet.

The main acute effects on metabolic processes were associated with diet composition. The change from diet D1 to D3 promoted reductions in both excreted N and respired C that were by 54% and 30% on average, respectively (i.e., computed for both conditioning diets and in F and S clams; Table 3).

The growth category (F vs. S) of clams conditioned to D1 exerted highly significant effects on all acquisition parameters, corresponding to the higher rates achieved by F compared to S clams (Table 5). However, the majority of these differences were observed in clams fed the D3 diet rather than the D1 diet (Figure 1), accounted for by a highly significant composition and growth category (C^*⁣*^*∗*^^G) interaction term for these parameters (Table 5). None of these growth category effects on the acquisition rates of nutrients were observed in clams conditioned to D3. Instead, metabolic losses of C and N were significantly higher in fast growers (Tables 4 and 6).

**Figure 3:**
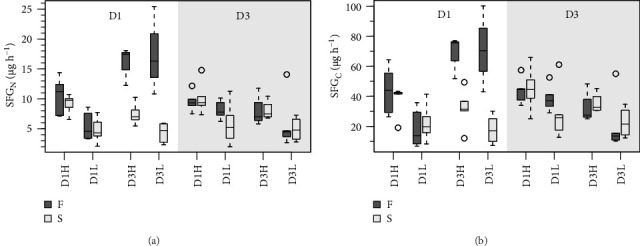
Net balances for N (a) and C (b) of F (dark boxes) and S (light boxes) clams subject to all nutritional conditions.

The resulting balances (SFG) of N and C are shown in Figure 3. The SFG values were consistently higher (~40%) in clams fed the high ration, although differences with the low ration were marginally significant (Tables 5 and 6). Additionally, food composition and growth condition exerted significant effects on N and C balances, but only in clams conditioned to D1. The most relevant effect was the large difference observed between F and S clams fed the D3 (Figure 3), accounted for by high significance of the C^*⁣*^*∗*^^G interaction (Table 5).

Food composition exerted significant effects on C:N ratios for all acquisition parameters, including AE (Tables 5 and 6). The C:N ratios for metabolic products and SFG were significantly affected by food composition but only in D3 conditioned clams. Extensive effects of ration on acquisition parameters were also recorded in this group, both as an isolated factor and in combination with food composition (C^*⁣*^*∗*^^R interaction; Table 6). Significant F vs. S differences were observed in the C:N ratio of certain acquisition parameters, particularly in D1 conditioning. However, C:N ratios for SFG exhibited significant differences between growth groups only after conditioning to D3.

## 4. Discussion

A combination of acute and chronic responses to diets differing in biochemical composition promoted differential effects on the acquisition and use of carbon (C) and nitrogen (N) in *R. decussatus* juveniles, despite the diets having similar C:N ratio. Additionally, these effects were dependent upon the ration dosed and differed between fast and slow growers. Overall, the main trends observed in elemental balances reflected the behavior observed in energy balances [18], although specific differences between the two approaches were reported for certain physiological components.

### 4.1. Optimal Energetic Status Enables Acute Feeding Responses and Nutritional Compensation

Given that C and N availability was similar across different dietary compositions, the IRs of both elements (IR_N_ and IR_C_) merely reflected the behavior of the CR (Table 2). The main evidence of an acute feeding response to changing diet composition was the pronounced increase in CR of clams conditioned to D1 when transferred to D3, particularly evident in F clams. As previously discussed [18], this would compensate for the drastic reduction in absorption efficiencies (AE_N_ and AE_C_) recorded in clams fed the D3 diet, allowing ARs to improve, on average, between exposure diets D1 and D3 (Table 4). In the absence of such overfeeding response, as seen in clams conditioned to D3, or S clams of the D1 conditioning group, the exposure to the low quality D3 diet resulted in significantly lower N and C ARs (Figure 1), translating to lower SFG values (Table 4). An increase in feeding activity to compensate for poorly digestible food items (more abundant in D3 diets) is a common response in bivalves [30]. However, the present results indicate that an optimal energetic status is a prerequisite for such an adjustment. This explains why this response was observed exclusively in the F clams from the D1 conditioning group. Clams of this same condition (F/D1) also compensated for the quantitative reduction in food supply, from high (H) to low (L) ration, by minimizing both N and C fecal losses, confirming the potential of prior nutritional status in active physiological regulation.

### 4.2. The Lipid-Poor D3 Diet Imposes Digestive Constraints That Reduce Elemental Balances

The digestive performances of clams fed two diets were neatly different as the absorption efficiencies of both N and C attained with the phytoplankton-rich diet (D1) were approximately 50% higher compared to the yeast-rich diet (D3). This difference was only slightly affected by the conditioning diet and growth category. Low susceptibility of yeast cells to the digestive enzyme attack in the clam gut, likely due to the unsuitable composition of their cell walls, was inferred from the strong negative relationship found with diet D3 for the overall organics AE (Conover) and the IR of organics [18]. This relationship also applies to the absorption processes of both elements (AE_N_ = 0.73 e^−13.99IR_N_^ and AE_C_ = 0.72 e^−3.34IR_C_^). Such a relationship indicates strong constraints on digestion and absorption processes, resulting in longer gut residence times for less digestive food particles [31]. This leads to greater digestive investments and enhanced losses of endogenous organics in the feces (i.e., the metabolic fecal losses: MFL) [4, 32]. Indeed, experimental evidence with bivalves fed on complex particle assemblages in seston shows that the digestive processing of more refractory organic particles (nonmicroalgal particles such as detritus) requires about twice the metabolic fecal losses per unit of organic ingestion compared to processing phytoplankton cells [33, 34].

**Figure 4:**
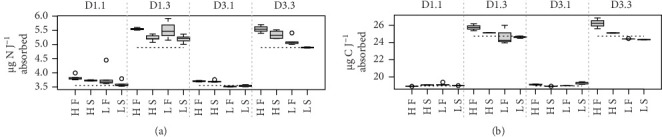
Rates of N (a) and C (b) absorption (µg) per Joule absorbed. Horizontal dotted lines represent rates of N and C (respectively) per Joule ingested.

Because MFLs are known to have an elevated lipid content, specific requirements for this component might be doubly constrained with diet D3. This is due to the poor digestive balances achieved with this diet, which has reduced lipid content and high rates of lipid egestion [18]. As a potentially limiting component, the digestive fate of lipids with the different diet compositions was indirectly assessed using a ratio of elements (N and C) to energy. This compared the amount (µg) of each element ingested or absorbed per Joule of energy ingested or absorbed, respectively (Figure 4). Due to the higher energy content of lipids compared to the rest of the major biochemical components (proteins and carbohydrates), the main difference in these ratios resulted from differences in food composition. The consistently lower values achieved with diet D1 reflected the higher lipid abundance characteristic of this phytoplankton-rich diet, compared to the lower energy content/higher abundance of carbohydrates in the yeast-rich diet (Figure 4). Likewise, departure of ratios computed for the absorbed ration from the corresponding values for the ingested ration (dashed lines in Figure 4) would indicate a reduction in the lipid content of feeds during digestive and absorptive processes. This is compatible with the reported lipid enrichment of fecal materials in bivalves [5, 35, 36], associated with MFL [4], which predominantly contain lysosomal membranes and lipid droplet residuals from intracellular digestion [37, 38]. This interpretation explains the higher digestive lipid imbalance found under the lipid-poor D3 diet, especially when dosed at high ration and in F clams (Figure 4). This is consistent with the concept that elevated digestive turnover rates and consequent high MFLs are hallmarks of these conditions.

**Figure 5:**
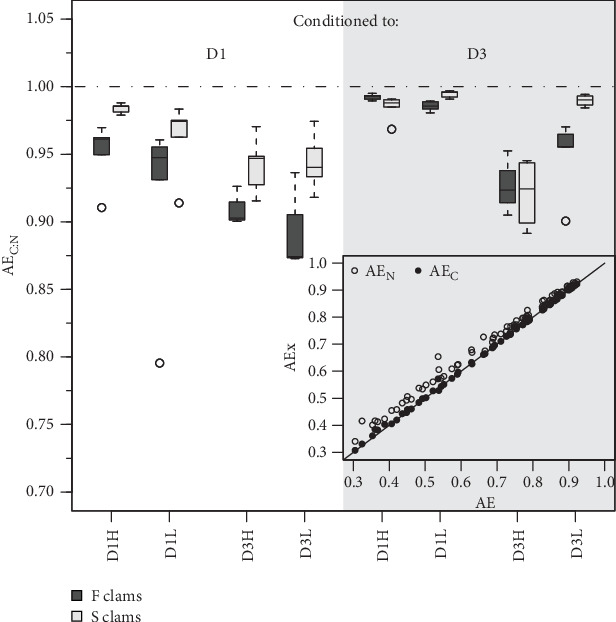
AE_C:N_ ratios of F (dark boxes) and S (light boxes) clams subject to the different nutritional condition. Inset: The relationship between absorption efficiency of each element (C and N) and overall organics absorption efficiency (Conover).

The differences in absorption vs. ingestion ratio were generally greater for N than for C (Figure 4), reflecting the assumedly low N content of MFLs in the form of a preferential absorption of N relative to C. The AE ratios for both elements (AE_C_/AE_N_ = AE_C:N_) were consistently below 1 (Figure 5), with notable quantitative differences between conditions. Overall, preferential N absorption (lower AE_C:N_ ratios) was greater in F clams and clams fed the low quality D3 diet at high ration, although this rule is exclusively applicable to the D1 conditioning group (Figure 5). The evidence presented in Figure 5 shows that the AE for N progressively diverges from that for C as the overall AE (Conover) decreases. This confirms the assumption that the preferential absorption of N (relative to C) would be quantitatively related to the degree of digestive performance constraint by the magnitude of MFL, as characteristically occurs with yeast-rich diets as well as diets dosed at high ration. Quite in the same line, Ibarrola et al. [39] reported differences in absorption efficiencies for major biochemical components in cockles (*C. edule*) that also increased with decreasing the AE of overall organics. The highest values were recorded for proteins and the lowest for lipids, while intermediate values were recorded for carbohydrates. The preferential absorption of N with low-quality diets promoting reduced AEs was a particularly remarkable feature of the digestive behavior in a congeneric clam species (*R. philippinarum*) fed microalgal cells manipulated to achieve an approximately 3x reduction in the N content relative to the high quality diet. In this case, AE of N exceeded that of C by up to 50% under chronic N deficit conditions [8]. The increased efficiency of N absorption associated with reduced dietary N availability was interpreted as a compensation for nutrient imbalances to maintain elemental homeostasis [3, 4, 6, 40–43]. This contrasts with the present experiments where no specific N limitation would be expected with the low-quality diets. However, the greater intensity of the digestive response observed under N deficit conditions suggests the involvement of additional active mechanisms (i.e., lysosomal protease induction [44]) to enhance protein absorption.

### 4.3. Postabsorptive Processes Significantly Influence Elemental Balances in Clams, With Notable Differences in C:N Ratios Driven by Diet Composition and Physiological Status

**Figure 6:**
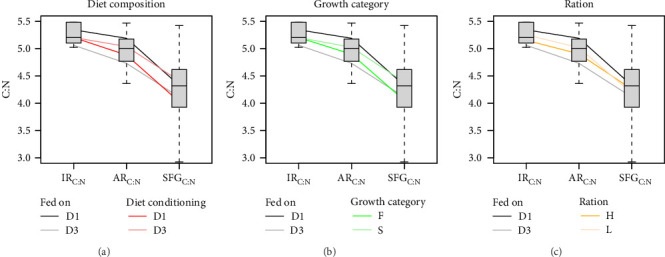
C:N ratios for IR, AR, and SFG. The lines represent the trajectory of each group combination: (a–c) The effects of feeding on either D1 (black) or D3 (gray). (a) The red lines depict the effects of conditioning, while (b) the green lines represent the effects of growth. (c) The orange lines illustrate the effects of rationing. Boxplots represent the mean of pooled values for each variable.

The release of metabolic products through respiration and ammonia excretion showed little variation among conditions and had minor effects on energy balances [18]. However, the combined contribution of both metabolic expenses to the net elemental balances, regarding C:N ratios, resulted crucial. When changes in these ratios are traced through the successive components of elemental balances (Figure 6), it becomes clear that the main step in the relative N enrichment achieved for the retained fraction (or SFG) occurs in the postabsorptive phase, primarily due to large amounts of C respired respect to the N excreted. On average, the contribution of these post-absorptive processes amounts to approximately 70% of total C:N change compared to approximately 30% attributable to digestive processes in the preabsorptive phase.

However, the noteworthy difference in both the extent of overall stoichiometric adjustments (total reduction of C:N ratios from the ingested to the retained fractions) and its distribution between pre- and postabsorptive components can be attributed to different growth or diet conditioning groups of clams. For instance, a more comprehensive adjustment was observed in F clams (Figure 6b; 21.5% mean reduction) compared to S clams (15.7%), as well as in clams conditioned to D1 (Figure 6a; 22.5%) compared to those conditioned to D3 (15.3%). No differences were observed in this respect between clams fed at high (16%) or low (18%) food rations (Figure 6c), and either D1 (19.0%) or D3 (18.5%) compositions, where initial differences in C:N ratios of the ingested diets were approximately maintained for the retained fraction. Thus, it appears that improved physiological status associated with both endogenous and nutritional conditions, prior to the onset of physiological measurements, enabled a more efficient stoichiometric coupling between consumed diets and biosynthetic requirements of growing tissues. With regard to the distribution of these adjustments between pre- and postabsorptive processes, no differences were observed between conditioning groups D1 and D3, which aligned with the mean 29%/71% distribution. Additionally, minimal deviations were noted for different growth groups, with metabolic processes having a slightly higher influence (73%) in S compared to F (69%) clams. However, great differences were observed related to the feeding regime during physiological experimentation. For instance, the fractional contribution changed from 12%/88% in clams fed the diet D1 to 45%/55% in those fed the diet D3. Clearly, the improved digestive balance of N achieved with the latter diet accounts for this greater contribution of preabsorptive processes towards the total stoichiometric adjustments.

In summary, an analysis of variations in the C:N ratio along the pre- and postabsorptive processes revealed significant differences in stoichiometric adjustments between clams fed diets with different L:CH ratios, despite similar C:N ratios in both diets. The lack of comparative N limitation suggests that the preferential absorption of this nutrient with the low-quality (low lipid) diet reflects greater digestive C imbalances, leading to considerable metabolic fecal losses. This lipid imbalance is likely to be the cause of the reduced growth rates observed in clams fed a less digestible diet, with AE declining to approximately 50% of the values recorded with the high-quality (high lipid) diet. The potential for overfeeding to compensate for reduced AE appears constrained by the clams' energetic status prior to the feeding experiments, a phenomenon observed only in fast-growing clams conditioned to high-quality (D1) diets.

## Figures and Tables

**Table 1:** Diet composition, carbohydrates (%), proteins (%), lipids (%), ratios of biochemical components (proteins/carbohydrates, Pr:CH; proteins/lipids, Pr:L; lipids/carbohydrates, L:CH) and elemental components, and energy content (E cont., J mg POM^−1^).

Diet	Composition	Carbohydrates	Proteins	Lipids	Pr:CH	Pr:L	L:CH	C:N	C:H	E cont.
D1	*R. lens*: 80% *S. cerevisiae*: 20%	15.47	50.33	34.20	3.25	1.47	2.21	4.77	4.15	26.29
D3	*R. lens*: 20% *S. cerevisiae*: 80%	32.60	47.85	19.55	1.48	2.44	0.60	4.84	3.71	19.96

**Table 2:** Mean (SD) size-standardized values of clearance rate (CR), absorption efficiency (AE), metabolic rate (VO_2_), and ammonia excretion rate (VNH_4_-N) obtained in Arranz et al. [18] and used here to calculate the elemental balances.

Conditioning diet	Exposure diet	Exposure ration	Growth	CR (L h^−1^)	AE	VO_2_ (µL h^−1^)	VNH_4_-N (µg h^−1^)
D1	D1	H	F	0.11 (0.04)	0.79 (0.08)	28.21 (4.41)	0.39 (0.09)
			S	0.09 (0.02)	0.89 (0.02)	32.41 (14.21)	0.44 (0.11)
		L	F	0.16 (0.06)	0.63 (0.18)	29.55 (6.33)	0.24 (0.12)
			S	0.12 (0.04)	0.73 (0.13)	13.84 (4.33)	0.3 (0.17)
	D3	H	F	0.32 (0.04)	0.34 (0.03)	19.07 (4.32)	0.18 (0.06)
			S	0.11 (0.04)	0.53 (0.1)	14.77 (16.79)	0.25 (0.18)
		L	F	0.39 (0.18)	0.57 (0.05)	25.3 (10.94)	0.3 (0.09)
			S	0.11 (0.04)	0.55 (0.11)	10.16 (5.7)	0.2 (0.12)

D3	D1	H	F	0.11 (0.02)	0.81 (0.05)	27.01 (10.67)	0.69 (0.3)
			S	0.1 (0.03)	0.88 (0.06)	27.45 (13.5)	0.47 (0.11)
		L	F	0.17 (0.03)	0.88 (0.03)	21.72 (8.91)	0.37 (0.12)
			S	0.14 (0.09)	0.84 (0.07)	14.87 (17.7)	0.43 (0.18)
	D3	H	F	0.14 (0.04)	0.4 (0.05)	17.19 (4.72)	0.22 (0.04)
			S	0.12 (0.02)	0.5 (0.09)	9.95 (6.32)	0.18 (0.07)
		L	F	0.12 (0.09)	0.67 (0.11)	22.76 (12.94)	0.15 (0.05)
			S	0.09 (0.03)	0.76 (0.08)	6.99 (0.92)	0.09 (0.03)

**Table 3:** Means of pooled values for the four factors (conditioning and exposure diets, ration, and growth category) regarding elemental balances parameters: ingestion (IR), egestion (ER), absorption (AR), ammonia excretion (E) and respiration (R) rates, and elemental balances (SFG) for N and C (µgh^−1^). Absorption efficiency (AE), as well as absorption efficiencies of C and N are given in decimal units.

Parameter	Conditioning diet		Exposure diet		Exposure ration		Growth category	
	D1	D3	D1	D3	H	L	F	S
IR_N_	14.28 (1.83)	10.91 (0.82)	10.08 (0.57)	15.75 (2.05)	15.82 (1.49)	9.55 (1.19)	15.48 (1.89)	10.02 (0.71)
IR_C_	73.76 (9.21)	56.8 (4.17)	53.59 (2.94)	79.89 (10.44)	81.42 (7.58)	50.02 (5.99)	80.09 (9.5)	52.03 (3.64)
ER_N_	5.4 (1.16)	2.93 (0.51)	1.69 (0.17)	7.26 (1.24)	5.75 (1.17)	2.66 (0.49)	5.63 (1.24)	2.85 (0.44)
ER_C_	30.59 (6.3)	16.25 (2.77)	10.16 (1.07)	39.99 (6.8)	31.77 (6.3)	15.52 (2.94)	31.85 (6.73)	15.88 (2.39)
AR_N_	8.88 (0.84)	7.99 (0.54)	8.39 (0.51)	8.49 (0.94)	10.07 (0.52)	6.89 (0.76)	9.85 (0.85)	7.17 (0.49)
AR_C_	43.17 (3.82)	40.55 (2.78)	43.43 (2.57)	39.89 (4.23)	49.64 (2.51)	34.5 (3.54)	48.25 (3.85)	36.15 (2.53)
AE	0.65 (0.03)	0.74 (0.03)	0.8 (0.02)	0.56 (0.03)	0.68 (0.04)	0.71 (0.02)	0.67 (0.03)	0.71 (0.03)
AE_N_	0.69 (0.03)	0.76 (0.03)	0.83 (0.02)	0.59 (0.02)	0.71 (0.03)	0.74 (0.02)	0.71 (0.03)	0.74 (0.03)
AE_C_	0.65 (0.03)	0.74 (0.03)	0.81 (0.02)	0.56 (0.03)	0.68 (0.04)	0.71 (0.02)	0.68 (0.03)	0.72 (0.03)
E_N_	0.31 (0.03)	0.36 (0.04)	0.44 (0.03)	0.2 (0.02)	0.4 (0.04)	0.28 (0.03)	0.35 (0.04)	0.32 (0.03)
R_C_	7.58 (0.7)	6.71 (0.73)	8.56 (0.66)	5.39 (0.66)	8 (0.74)	6.34 (0.67)	8.58 (0.53)	5.87 (0.78)
SFG_N_	8.58 (0.84)	7.62 (0.52)	7.95 (0.49)	8.29 (0.94)	9.67 (0.52)	6.61 (0.76)	9.5 (0.85)	6.85 (0.48)
SFG_C_	35.59 (3.7)	33.84 (2.51)	34.87 (2.46)	34.51 (3.98)	41.64 (2.5)	28.16 (3.3)	39.67 (3.75)	30.28 (2.35)

**Table 4:** Mean (SD) values of physiological responses of F and S clams conditioned to either D1 or D3 then fed on either D1 or D3: ingestion (IR), egestion (ER), absorption (AR), ammonia excretion (E) and respiration (R) rates, and elemental balances (SFG) for N and C (µgh^−1^). I_C:N_, E_C:N_, A_C:N_, and M_C:N_ stand for the C:N ratio of the ingested, egested, and absorbed materials, and the C:N of metabolic losses (R_C_/E_N_), respectively.

Diet growth	D1		D3		Statistical differences		
	F	S	F	S	D	G	D^*⁣*^*∗*^^G
Exposure to D1							
IR_N_	13.21 (4.14)^D^	10.6 (1.77)	12.45 (1.96)	11.99 (3.12)	—	—	—
IR_C_	68.74 (21.54)^D^	55.18 (9.23)	64.82 (10.19)	62.39 (16.22)	—	—	—
ER_N_	2.32 (1.34)^D,G^	1 (0.28)	2.16 (0.64)	1.32 (0.76)	—	*⁣* ^*∗*^	—
ER_C_	14.97 (8.61)^D,G^	6.04 (1.71)	11.67 (3.45)	7.74 (4.43)	—	*⁣* ^*∗*^	—
AR_N_	10.88 (3.2)	9.6 (1.57)	10.29 (1.69)	10.66 (2.81)	—	—	—
AR_C_	53.77 (15.7)	49.14 (7.99)	53.15 (8.78)	54.64 (14.51)	—	—	—
AE_N_	0.83 (0.06)^D,G^	0.91 (0.02)	0.83 (0.04)	0.89 (0.06)	—	*⁣* ^*∗∗*^	—
AE_C_	0.79 (0.08)^D,G^	0.89 (0.02)	0.82 (0.04)	0.88 (0.06)	—	*⁣* ^*∗∗*^	—
E_N_	0.41 (0.09)	0.46 (0.12)	0.73 (0.32)	0.49 (0.11)	—	—	—
R_C_	9.9 (1.55)	11.38 (4.99)	9.48 (3.75)	9.64 (4.74)	—	—	—
SFG_N_	10.47 (3.2)	9.13 (1.65)	9.56 (1.71)	10.17 (2.81)	—	—	—
SFG_C_	43.86 (16.38)	37.76 (10.45)	43.67 (8.99)	45 (15.05)	—	—	—
I_C:N_	5.21 (0.02)^D^	5.21 (0.02)	5.21 (0.02)	5.21 (0.02)	—	—	—
E_C:N_	6.44 (0.03)^D,G,^⁣^D^*∗*^^^G^	6.01 (0.04)	5.39 (0)	5.85 (0)	*⁣* ^*∗∗∗*^	*⁣* ^*∗∗∗*^	*⁣* ^*∗∗∗*^
A_C:N_	4.95 (0.12)^D^	5.12 (0.02)	5.17 (0.01)	5.12 (0.05)	*⁣* ^*∗∗*^	*⁣* ^*∗*^	*⁣* ^*∗∗*^
AE_C:N_	0.95 (0.02)^D^	0.98 (0.00)	0.99 (0)	0.98 (0.01)	*⁣* ^*∗∗*^	*⁣* ^*∗*^	*⁣* ^*∗∗*^
M_C:N_	25.49 (8)	25.48 (12.28)	14.74 (8.08)	20.49 (11.03)	—	—	—
SFG_C:N_	4.12 (0.34)	4.08 (0.71)	4.56 (0.36)	4.37 (0.64)	—	—	—
Exposure to D3							
IR_N_	41.54 (4.71)	14.25 (4.54)	18.45 (4.85)	15.29 (2.88)	*⁣* ^*∗∗*^	*⁣* ^*∗∗∗*^	*⁣* ^*∗∗∗*^
IR_C_	212 (24.02)	72.7 (23.19)	94.14 (24.74)	78.02 (14.71)	*⁣* ^*∗∗*^	*⁣* ^*∗∗∗*^	*⁣* ^*∗∗∗*^
ER_N_	25.45 (1.65)	6.5 (2.97)	10.02 (1.78)	7.05 (2.19)	*⁣* ^*∗∗∗*^	*⁣* ^*∗∗∗*^	*⁣* ^*∗∗∗*^
ER_C_	137.2 (10.06)	35.56 (16.26)	54.51 (11.22)	39.25 (12.19)	*⁣* ^*∗∗*^	*⁣* ^*∗∗∗*^	*⁣* ^*∗∗∗*^
AR_N_	16.1 (3.25)	7.74 (1.87)	8.43 (3.17)	8.23 (1.57)	—	*⁣* ^*∗∗*^	*⁣* ^*∗∗*^
AR_C_	74.79 (15.45)	37.14 (8.77)	39.63 (13.94)	38.76 (7.93)	—	*⁣* ^*∗∗*^	*⁣* ^*∗*^
AE_N_	0.38 (0.04)	0.56 (0.1)	0.45 (0.05)	0.54 (0.08)	—	*⁣* ^*∗∗*^	—
AE_C_	0.35 (0.04)	0.53 (0.1)	0.42 (0.04)	0.5 (0.09)	—	*⁣* ^*∗∗*^	—
E_N_	0.19 (0.07)	0.27 (0.19)	0.24 (0.04)	0.19 (0.08)	—	—	—
R_C_	6.69 (1.52)	5.19 (5.89)	6.04 (1.66)	3.49 (2.22)	—	—	—
SFG_N_	15.91 (3.19)	7.47 (1.82)	8.19 (3.15)	8.04 (1.64)	—	*⁣* ^*∗∗*^	*⁣* ^*∗∗*^
SFG_C_	68.1 (14.15)	31.95 (13.45)	33.59 (12.8)	35.27 (6.86)	—	*⁣* ^*∗*^	*⁣* ^*∗*^
I_C:N_	5.1 (0.03)	5.1 (0.03)	5.1 (0.03)	5.1 (0.03)	—	—	—
E_C:N_	5.39 (0.05)	5.47 (0)	5.42 (0.16)	5.56 (0.10)	—	*⁣* ^*∗*^	—
A_C:N_	4.64 (0.07)	4.81 (0.11)	4.73 (0.12)	4.7 (0.13)	—	—	—
AE_C:N_	0.91 (0.01)	0.94 (0.02)	0.93 (0.02)	0.92 (0.03)	—	—	—
M_C:N_	37.31 (6.38)	36.99 (62.14)	25.89 (4.14)	26.2 (26.51)	—	—	—
SFG_C:N_	4.27 (0.05)	4.13 (1.08)	4.11 (0.19)	4.4 (0.3)	—	—	—

*Note*: Results of two-way ANOVA *p*-values for comparisons between acclimation effects are also shown; where D and G represent diet and growth group effect, respectively. Asterisks display different significance levels (*⁣*^*∗*^*p*  < 0.05, *⁣*^*∗∗*^*p*  < 0.01; *⁣*^*∗∗∗*^*p*  < 0.001). Superscripts (D, G, and D*⁣*^*∗*^G) indicate differences between conditioning groups (D1 and D3) when they are fed on their conditioning diet.

**Table 5:** Three-way ANOVA main results for elemental balances parameters, employing composition and ration of exposure diet, and growth category as factors for clams conditioned to D1.

Parameter	Composition (C)	Ration (R)	Growth category (G)	C:R	C:G	R:G	C:R:G
**IR_N_**	***F* = 38.853, **p** < 0.001**	***F* = 15.741, **p** < 0.001**	***F* = 49.325, **p** < 0.001**	*F* = 2.214, *p* = 0.148	***F* = 42.316, **p** < 0.001**	*F* = 1.014, *p* = 0.322	*F* = 0.876, *p* = 0.357
**IR_C_**	***F* = 34.826, **p** < 0.001**	***F* = 15.302, *p* = 0.001**	***F* = 49.061, **p** < 0.001**	*F* = 2.757, *p* = 0.108	***F* = 41.168, **p** < 0.001**	*F* = 1.062, *p* = 0.312	*F* = 0.992, *p* = 0.328
**ER_N_**	***F* = 107.635, **p** < 0.001**	***F* = 17.781, **p** < 0.001**	***F* = 70.134, **p** < 0.001**	***F* = 30.514, **p** < 0.001**	***F* = 58.418, **p** < 0.001**	***F* = 11.169, **p** = 0.002**	***F* = 15.01, **p** = 0.001**
**ER_C_**	***F* = 88.82, **p** < 0.001**	***F* = 13.267, **p** = 0.001**	***F* = 66.438, **p** < 0.001**	***F* = 23.826, **p** < 0.001**	***F* = 50.656, **p** < 0.001**	***F* = 7.743, **p** = 0.01**	***F* = 10.178, **p** = 0.003**
**AR_N_**	***F* = 5.441, **p** = 0.027**	***F* = 10.598, **p** = 0.003**	***F* = 25.883, **p** < 0.001**	*F* = 2.903, *p* = 0.099	***F* = 23.006, **p** < 0.001**	*F* = 0.737, *p* = 0.398	*F* = 1.872, *p* = 0.182
**AR_C_**	*F* = 2.45, *p* = 0.129	***F* = 11.755, **p** = 0.002**	***F* = 21.565, **p** < 0.001**	*F* = 2.579, *p* = 0.12	***F* = 20.927, **p** < 0.001**	*F* = 0.594, *p* = 0.447	*F* = 1.449, *p* = 0.239
**AE_N_**	***F* = 56.235, **p** < 0.001**	*F* = 1.266, *p* = 0.27	***F* = 4.714, **p** = 0.039**	***F* = 17.793, **p** < 0.001**	*F* = 0.034, *p* = 0.854	*F* = 2.335, *p* = 0.138	*F* = 3.251, *p* = 0.082
**AE_C_**	***F* = 47.218, **p** < 0.001**	*F* = 1.698, *p* = 0.203	***F* = 6.44, **p** = 0.017**	***F* = 13.818, **p** = 0.001**	*F* = 0.046, *p* = 0.832	*F* = 1.441, *p* = 0.24	*F* = 1.831, *p* = 0.187
***E*_N_**	***F* = 6.543, **p** = 0.016**	*F* = 3.228, *p* = 0.083	*F* = 0.369, *p* = 0.548	*F* = 3.292, *p* = 0.08	*F* = 0.564, *p* = 0.459	*F* = 0.609, *p* = 0.442	*F* = 1.215, *p* = 0.28
***R*_C_**	***F* = 9.172, **p** = 0.005**	*F* = 2.401, *p* = 0.132	***F* = 5.127, **p** = 0.031**	*F* = 1.527, *p* = 0.227	*F* = 0.356, *p* = 0.556	***F* = 5.921, **p** = 0.022**	*F* = 0.464, *p* = 0.501
**SFG_N_**	***F* = 6.144, **p** = 0.019**	***F* = 10.324, **p** = 0.003**	***F* = 26.811, **p** < 0.001**	*F* = 2.695, *p* = 0.112	***F* = 23.242, **p** < 0.001**	*F* = 0.695, *p* = 0.412	*F* = 1.781, *p* = 0.193
**SFG_C_**	***F* = 5.061, **p** = 0.033**	***F* = 9.056, **p** = 0.005**	***F* = 16.299, **p** < 0.001**	*F* = 1.664, *p* = 0.208	***F* = 19.01, **p** < 0.001**	*F* = 0.035, *p* = 0.853	*F* = 1.806, *p* = 0.19
**IR_C:N_**	***F* = 2.4 10** **^29^** **, **p** < 0.001**	***F* = 4.5 10** **^28^** **, **p** < 0.001**	*F* = 1.79, *p* = 0.192	***F* = 9.9 10** **^28^** **, **p** < 0.001**	*F* = 0.507, *p* = 0.482	*F* = 2.275, *p* = 0.143	*F* = 0.768, *p* = 0.388
**ER_C:N_**	***F* = 721.218, **p** < 0.001**	*F* = 3.65, *p* = 0.066	***F* = 131.788, **p** < 0.001**	***F* = 21.804, **p** < 0.001**	*F* = 2.896, *p* = 0.1	***F* = 9.28, **p** = 0.005**	***F* = 68.206, **p** < 0.001**
**AR_C:N_**	***F* = 43.667, **p** < 0.001**	*F* = 0.174, *p* = 0.68	***F* = 11.553, **p** = 0.002**	*F* = 3.205, *p* = 0.084	*F* = 0, *p* = 0.998	*F* = 0.387, *p* = 0.539	*F* = 0.005, *p* = 0.943
**AE_C:N_**	***F* = 5.375, **p** = 0.0280**	*F* = 2.346, *p* = 0.14	***F* = 12.216, **p** = 0.002**	*F* = 1.058, *p* = 0.312	*F* = 0.01, *p* = 0.92	*F* = 0.364, *p* = 0.55	*F* = 0.023, *p* = 0.881
***M*_C:N_**	*F* = 0.055, *p* = 0.816	*F* = 0.213, *p* = 0.648	*F* = 1.316, *p* = 0.261	*F* = 1.585, *p* = 0.218	*F* = 0.74, *p* = 0.397	*F* = 1.288, *p* = 0.266	*F* = 0.762, *p* = 0.39
**SFG_C:N_**	*F* = 0.36, *p* = 0.554	*F* = 1.132, *p* = 0.296	*F* = 1.772, *p* = 0.194	*F* = 0.052, *p* = 0.821	*F* = 2.686, *p* = 0.112	*F* = 2.883, *p* = 0.101	*F* = 2.083, *p* = 0.16

*Note*: Results in bold indicate *p* values lower than 0.05 and thus considered as significant differences between groups.

**Table 6:** Three-way ANOVA main results for elemental balances parameters, employing composition and ration of exposure diet, and growth category as factors for clams conditioned to D3.

Parameter	Composition (C)	Ration (R)	Growth category (G)	C:R	C:G	R:G	C:R:G
**IR_N_**	*F* = 0.858, *p* = 0.362	***F* = 22.319, **p** < 0.001**	*F* = 1.652, *p* = 0.209	***F* = 4.714, **p** = 0.039**	*F* = 0.289, *p* = 0.595	*F* = 0.022, *p* = 0.883	*F* = 0.234, *p* = 0.632
**IR_C_**	*F* = 0.27, *p* = 0.607	***F* = 20.431, **p** < 0.001**	*F* = 1.638, *p* = 0.211	***F* = 5.458, **p** = 0.027**	*F* = 0.242, *p* = 0.626	*F* = 0.029, *p* = 0.866	*F* = 0.259, *p* = 0.615
**ER_N_**	***F* = 61.064, **p** < 0.001**	***F* = 53.266, **p** < 0.001**	*F* = 3.501, *p* = 0.072	***F* = 46.005, **p** < 0.001**	*F* = 3.728, *p* = 0.064	*F* = 3.038, *p* = 0.092	*F* = 0.165, *p* = 0.688
**ER_C_**	***F* = 54.977, **p** < 0.001**	***F* = 48.681, **p** < 0.001**	*F* = 3.091, *p* = 0.09	***F* = 43.723, **p** < 0.001**	*F* = 3.523, *p* = 0.071	*F* = 1.94, *p* = 0.175	*F* = 0.106, *p* = 0.747
**AR_N_**	***F* = 4.922, **p** = 0.035**	***F* = 9.061, **p** = 0.005**	*F* = 0.756, *p* = 0.392	*F* = 0.016, *p* = 0.901	*F* = 0.02, *p* = 0.888	*F* = 0.926, *p* = 0.344	*F* = 0.21, *p* = 0.65
**AR_C_**	***F* = 8.9, **p** = 0.006**	***F* = 6.988, **p** = 0.013**	*F* = 0.734, *p* = 0.399	*F* = 0.016, *p* = 0.899	*F* = 0.072, *p* = 0.791	*F* = 0.85, *p* = 0.364	*F* = 0.276, *p* = 0.603
**AE_N_**	***F* = 112.266, **p** < 0.001**	***F* = 25.045, **p** < 0.001**	*F* = 2.151, *p* = 0.154	***F* = 25.346, **p** < 0.001**	*F* = 2.089, *p* = 0.159	*F* = 3.114, *p* = 0.089	*F* = 0.629, *p* = 0.434
**AE_C_**	***F* = 114.736, **p** < 0.001**	***F* = 24.85, **p** < 0.001**	*F* = 2.469, *p* = 0.127	***F* = 25.158, **p** < 0.001**	*F* = 2.484, *p* = 0.126	*F* = 1.317, *p* = 0.261	*F* = 0.873, *p* = 0.358
**E_N_**	***F* = 43.793, **p** < 0.001**	***F* = 7.177, **p** = 0.012**	*F* = 1.899, *p* = 0.179	*F* = 0.862, *p* = 0.361	*F* = 0.096, *p* = 0.759	*F* = 2.483, *p* = 0.126	*F* = 2.357, *p* = 0.136
**R_C_**	***F* = 4.652, **p** = 0.04**	*F* = 1.012, *p* = 0.323	*F* = 3.873, *p* = 0.059	*F* = 1.971, *p* = 0.171	*F* = 1.345, *p* = 0.256	*F* = 1.06, *p* = 0.312	*F* = 0.006, *p* = 0.937
**SFG_N_**	*F* = 3.486, *p* = 0.072	***F* = 8.298, **p** = 0.008**	*F* = 0.638, *p* = 0.431	*F* = 0.006, *p* = 0.941	*F* = 0.016, *p* = 0.901	*F* = 1.113, *p* = 0.300	*F* = 0.298, *p* = 0.59
**SFG_C_**	***F* = 6.557, **p** = 0.016**	***F* = 6.412, **p** = 0.017**	*F* = 0.118, *p* = 0.734	*F* = 0.074, *p* = 0.788	*F* = 0.388, *p* = 0.538	*F* = 0.47, *p* = 0.498	*F* = 0.342, *p* = 0.563
**IR_C:N_**	***F* = 1.2 10^30^, **p** < 0.001**	***F* = 2.1 10^29^, **p** < 0.001**	***F* = 1.8 10^27^, **p** < 0.001**	***F* = 4.5 10^29^, **p** < 0.001**	*F* = 0.003, *p* = 0.958	*F* = 3.748, *p* = 0.063	*F* = 2.433, *p* = 0.13
**ER_C:N_**	***F* = 433.999, **p** < 0.001**	***F* = 69.35, **p** < 0.001**	***F* = 26.568, **p** < 0.001**	***F* = 164.463, **p** < 0.001**	***F* = 27.38, **p** < 0.001**	***F* = 706.251, **p** < 0.001**	***F* = 43.33, **p** < 0.001**
**AR_C:N_**	***F* = 340.054, **p** < 0.001**	***F* = 70.749, **p** < 0.001**	*F* = 3.738, *p* = 0.063	***F* = 6.17, **p** = 0.019**	*F* = 3.395, *p* = 0.076	***F* = 8.022, **p** = 0.008**	*F* = 1.75, *p* = 0.197
**AE_C:N_**	***F* = 60.844, **p** < 0.001**	***F* = 14.648, **p** < 0.001**	*F* = 2.290, *p* = 0.141	***F* = 16.694, **p** < 0.001**	*F* = 3.574, *p* = 0.069	***F* = 7.840, **p** = 0.009**	*F* = 1.916, *p* = 0.177
**M_C:N_**	***F* = 6.493, **p** = 0.017**	*F* = 1.343, *p* = 0.256	*F* = 1.702, *p* = 0.203	*F* = 1.776, *p* = 0.193	*F* = 1.082, *p* = 0.307	*F* = 2.215, *p* = 0.148	*F* = 0.33, *p* = 0.57
**SFG_C:N_**	***F* = 11.051, **p** = 0.002**	*F* = 0.333, *p* = 0.568	***F* = 4.965, **p** = 0.034**	*F* = 4.188, *p* = 0.05	*F* = 2.507, *p* = 0.125	*F* = 3.442, *p* = 0.074	*F* = 0.038, *p* = 0.848

*Note*: Results in bold indicate *p* values lower than 0.05 and thus considered as significant differences between groups.

## Data Availability

The data that support the findings of this study are openly available in Zenodo at https://doi.org/10.5281/zenodo.14035420.
